# Occupational Exposure to Respirable Dust, Crystalline Silica and Its Pulmonary Effects among Workers of a Cement Factory in Kermanshah, Iran

**Published:** 2019-02

**Authors:** Ali Omidianidost, Sasan Gharavandi, Mansour R. Azari, Amir Hossein Hashemian, Mehdi Ghasemkhani, Fatemeh Rajati, Mehdi Jabari

**Affiliations:** 1 Research Center for Environmental Determinants of Health (RCEDH), Kermanshah University of Medical Sciences, Kermanshah, Iran,; 2 Department of Occupational Health, School of Public Health and Safety, Shahid Beheshti University of Medical Sciences, Tehran, Iran,; 3 Department of Occupational Health Engineering, School of Public Health, Tehran University of Medical Sciences, Tehran, Iran

**Keywords:** Cement dust, Respirable dust, Crystalline silica, Pulmonary function

## Abstract

**Background:**

Although the main hazard in cement processing is dust, and its effects on pulmonary function constitute the most important group of occupational diseases in this industry, evidence for association between exposure to cement dust and pulmonary function has not been conclusive. This study was performed with the aim to evaluate the impact of cement dust in the workplace on decreasing pulmonary function parameters among the workers.

**Materials and Methods:**

In this cross-sectional study 283 workers were studied, of which 140 workers were considered as exposed group and 143 workers as non-exposed group. Fifty samples of respirable dust were collected from breathing zone of workers in different sections of cement factory. Visible absorption spectrophotometry was used according to the NIOSH Method 7601 to measure crystalline silica content of reparable dust samples. Spirometry test was also applied to assess workers’ pulmonary function parameters.

**Results:**

Respirable dust concentration was in the range of 1.77 to 6.12 mg/m^3^. The concentration of crystalline silica in all units was higher than the Threshold Limit Value (TLV) (0.025 mg / m^3^). There were a significant difference in the Peak Expiratory Flow (PEF) parameter among workers in the two exposed and non-exposed groups to respirable dust (P= 0.017). In other parameters of pulmonary function (FVC, FEV_1_, FEF _25–75_, FEV_1_/FVC %), there were no significant differences between the two groups under study (P= 0.45, P= 0.14, P= 0.29 and P= 0.23, respectively).

**Conclusion:**

The findings of this study have provided an evidence to confirm the hypothesis that exposure to cement dust can cause complication in PEF parameter of cement industry workers.

## INTRODUCTION

Work environments have the potential harm to workers. People, who are working in industrial environments, may be exposed to dust inhalation risk (1). Dust contains crystalline silica as a known inhalation risk (2). Cement manufacturing plants are one of the most important manufacturing industries where the workers are exposed to dust in the different production sectors and processes. Cement dust aerodynamic diameter is in the range of respirable dust which has the ability to influence and deposit in the alveoli (3). Portland cement inhalation exposure is associated with the incidence of respiratory disease and varying degrees of airflow obstruction. The risk of respiratory diseases for workers in these industries is high. These diseases include asthma, chronic bronchitis and silicosis (4).

Cement is produced through a series of processes, including crushing, material handling, mixing and melting raw materials to produce clinker, cement mill and packing. A lot of dust is emitted from various parts of the production process that workers in these industries are at risk of exposure to it (5, 6). Portland cement is a mixture of calcium oxide, aluminum trioxide, silicon dioxide, ferric oxide, di-calcium silicate, magnesium oxide, selenium, thallium, and small amounts of hexavalent chromium. Cement plants’ workers face with numerous health risks during production and use of cement, including cement dust (7). Respirable dust can penetrate different parts of the respiratory system from the nose and mouth to bronchial and alveolar regions and get deposited there (8). Crystalline silica is another risk factor for lung disease which is found in cement dust, and exposure to it can cause many diseases including silicosis, lung cancer and lung airways disease.

There is conflicting evidence on the relationship between exposure to cement dust and respiratory symptoms or lung disorders. Several studies have confirmed the relationship between exposure to cement dust in cement production factories and its chronic effects on respiratory symptoms and pulmonary function (9–14), while other studies have not confirmed the relationship between exposure to cement dust and lung diseases (15–17).

Aminian et al. during a conducted study on 200 workers of a cement factory showed that the average concentration of total dust in the exposed group was 16.55 mg/m^3^. Results of the study showed that there was a significant decrease in the values of Peak Expiratory Flow (PEF), Forced Expiratory Volume in one second (FEV_1_), Forced Expiratory Flow (FEF) 25–75, Forced Vital Capacity (FVC) and FEV_1_/FVC in the exposed group compared to the control group (7). The results of a study conducted by Haider Noori et al. showed that in a cement factory with 180 workers, there was a significant reduction in the FEV1 and FEV1/FVC ratio compared with the control group. Lung function indices did not show a significant relationship between exposure to cement dust and smoking (8). The study conducted by Meo et al. showed that there was a significant reduction in mean pulmonary volumes including: FEV1, FVC and PEF in workers who were exposed to the cement dust for more than 10 years as compared to the control group (14).

According to the information referred to above, it seems that cement dust has the potential to harm lung function, but there is conflicting information and with regard to this matter, in order to prove this, more research should be done.

This study was performed with the aim to evaluate the impact of cement dust in the workplace on decreasing pulmonary function parameters among the workers.

## MATERIALS AND METHODS

### The study area and the production process

This study was a cross-sectional research conducted in a cement plant in the city of Kermanshah located in Iran. Six hundred workers are working in this factory during two work shifts. Raw materials needed included ironstone, silica, clay, limestone, gypsum. It is composed of several units including: crusher, soil hall, raw materials mill, cement mill, packing and loading. In the crushing unit and soil hall, raw materials become smaller in size, as well as the requirements of each of the raw materials are weighed and mixed together. From this point on, the raw materials are transferred to the mill, and then melted in the material furnace, they are converted to the clinker, and then transferred to cement mill, where some gypsum is added to it. Then the cement produced is transmitted to the packaging unit.

### Evaluation of the dust exposure

A total number of 6 Units were assessed, of which 50 samples of dust were collected from the breathing zone of workers in different parts of the plant. Sampling was done using a personal sampling pump, DELOXE model, SKC Company, HD, Mixed Cellulose Ester (MCE) filter with size of 0.8 microns, a diameter of 37 mm and with flow rate of 2.2 liters per minute (18). We used the NIOSH-0600 method as gravimetric to determine the concentration of respirable dust (19), and used the NIOSH 7601 method and visible absorption spectrophotometry at wavelengths of 420 and 820 nm (18).

### Study and samples design

Considering the 95% confidence, 80% test power and the formula for sample size estimate, the minimum sample size was estimated to be 6 in each group, but to increase reliability and accuracy, all the subjects were checked in. Therefore, 140 employees of the production line were selected as the exposed group and 143 office workers were selected as the non- exposed group. In order to evaluate pulmonary function and lung capacity of workers, required tests including measuring values FVC, FEV_1_, FEV_1_/FVC ratio, PEF and “forced expiratory flow over the middle half of the FVC” (FEF 25–75%) were performed at the end of the work shift, using Spirometry (Model SPIROLAB III made by Italian company MIR S.R.L), as recommended by the Thoracic Society of America (ATS) (20). The results of each test were interpreted by physician specializing in occupational medicine.

### Statistical tests

SPSS version 16 statistical software was used to analyze the data. The values of quantitative variables were evaluated using Kolmogorov test. The variables with normal distribution were compared using T-test and the rest were compared by Mann-Whitney test in two groups.

### Ethical considerations

The protocol of the study was approved by the Ethics Committee of Kermanshah University of Medical Sciences.

## RESULTS

The average age of the workers was 35.73±73 and 35.23±23 years in the exposed and non- exposed groups, respectively. There were no significant difference between the two groups (P=0.132). The average work experience for all subjects in two groups were five years, in fact, since the factory is newly established and has begun its activity for five years, so all personnel have been employed since the establishment of the factory during the past five years. The mean weight in the exposed and non-exposed groups was 79.78±13.2 82.5±12 kg, respectively, There were no significant difference between the two groups (P=0.071). The mean height in the exposed group was 176.13±6.7 and in the non-exposed was 176.76±6 cm, respectively. There were no significant difference between the two groups (P=0.2). 13% of the exposure group and 17% of the non-exposure group were smokers, but there were no significant differences in the use of cigarette between the exposed and non-exposed groups (P =0.39).

Based on the result of the exposure of workers to respirable dust and crystalline silica in different units, respirable dust concentration was in the range of 1.77 to 6.12 mg/m^3^. Exposure to respirable dust in the crusher unit was higher than other units. Respirable dust concentration in the office unit was the lowest. Crystalline silica exposure in the raw mill unit was higher than other units (Table 1). The concentration of crystalline silica in all units was higher than the Threshold Limit Value (TLV) (0.025 mg/m^3^).

**Table 1.** Mean of crystalline silica and respirable dust in the various casting processes (mg/m^3^)

**Units**	**Crystalline silica (mg/m^3^)**	**Respirable dust (mg/m^3^)**
Crusher	0.035±0.012	6.12±3.99
Packing and Loading	0.027±0.06	3.4±0.84
Cement mill	0.039±0.054	1.77±0.24
Raw mill	0.044±0.058	2.92±1.14
Stacker and reclaimer	0.026±0.005	2.52±0.85
Administration	-	0.8± (0.7)

According to the results of lung function tests, there were a significant difference in the PEF parameter among workers in two exposed and non-exposed groups to respirable dust (P= 0.017, Table 2). In other parameters of pulmonary function (FVC, FEV_1_, FEF_25–75_, FEV_1_/FVC %), there were no significant differences between the two exposed and non-exposure groups (P= 0.45, P= 0.14, P= 0.29 and P= 0.23 respectively; Table 2).

**Table 2.** Pulmonary function means in exposed and non– exposed groups

**Lung function tests**	**Exposed**	**Non-exposed**	**P-value**
FVC(L)	4.8±0.73	4.88±0.75	0.45
FEV_1_(L)	3.88±0.63	3.98±0.68	0.14
FEF_25–75_%(L/S)	3.98±1.05	4.06±0.86	0.29
FEV_1_/FVC (%)	91.9±9.9	93.2±9.8	0.23
PEF(L/S)	10.04±1.3	10.4±1.2	0.017^*^

## DISCUSSION

In the present study, there were no significant differences in regard to demographic data and smoking cigarettes between the exposure and non-exposure groups. In addition, the workers were monitored for exposure to respirable dust and crystalline silica. And their correlation with the lung function test results was investigated. For this purpose, workers were divided into two groups of exposed and non-exposed to cement dust.

Exposure to respirable dust concentrations was in the range of 1.77 to 6.12 mg/m^3^. And crystalline silica exposure was in the range of 0.026 to 0.044 mg/m^3^. Almost all workers had higher exposure to crystalline silica and respirable dust than the TLV by the American Conference Governmental Industrial Hygienists (ACGIH) (0.025 mg/m^3^, 1 mg/m^3^ respectively), except in administrative units (21, 22). Based on the results of a study carried out in Jordan in a cement industry, occupational exposure of workers of the packer unit to respirable dust were 3.9 mg/m^3^, which was similar to the results of the present study (23). However, occupational exposure of cement factory workers in our research was lower than exposures in recent studies on Mashhad and Ardebil Cement factory workers (24, 25).

According to the results, 95% of the exposed workers had normal respiratory functions, while 1% demonstrated obstructive and 4% restrictive lung function complications. The mean lung function parameter, including PEF, was significantly more than among exposed workers, compared to the non-exposure group. The mean lung function parameters, including; FVC, FEV_1_, FEF_25–75_% and FEV_1_/FVC%, between the two groups were not statistically significant. However, the mean of all parameters of pulmonary function in the exposed group was higher than the non-exposed group.

In studies conducted in Iraq and Ethiopia, similar results were obtained in which lung function parameter (PEF) in exposed group showed a significant decrease compared to non-exposure group, which was in agreement with the results of this study (26, 27). The results obtained in our study were different from the results of the Tavakol et al. study, in which the mean of %FEV_1_ and %FVC had a significant difference among all construction groups and the controls (28). In a study which was conducted by Kakooei et al. in a cement factory, the variables FVC, FEV1 and PEF showed significant reduction in the exposed group compared to the non-exposed group, the results of which were slightly different from this study (29). Kumar et al. concluded that the value of FEV_1_ in workers exposed to cement dust is normal, or is decreased the same as decreasing in the FVC, and thus, FEV_1_/FVC remains close to the normal value, which is consistent with the current study (30).

In studies on the effects of occupational exposure to cement dust on pulmonary function, there are conflicting results. The main reason for these contradictions is unclear, but it seems that differences such as dust concentration in the workplace, duration of exposure to dust, and the proper use of appropriate protective equipment, may be partly the reasons for these contradictions.

## CONCLUSION

The results of this study indicate that cement dust containing crystalline silica can cause respiratory problems especially a significant difference in the PEF parameter in the two exposed and non-exposed groups of workers. So, to prevent the development of lung diseases and respiratory disorders, especially in units with high dust concentrations, it is essential that the necessary measures be taken in this regard using engineering techniques and effective use of appropriate respiratory masks.
